# Announcing the *Journal of the Medical Library Association*’s data sharing policy

**DOI:** 10.5195/jmla.2019.801

**Published:** 2019-10-01

**Authors:** Katherine G. Akers, Kevin B. Read, Liz Amos, Lisa M. Federer, Ayaba Logan, T. Scott Plutchak

**Affiliations:** Editor-in-Chief, *Journal of the Medical Library Association*, JMLA@journals.pitt.edu; Data Services Librarian and Data Discovery Lead, NYU Health Sciences Library, New York University School of Medicine, New York, NY, kevin.read@nyumc.org; Librarian, National Information Center on Health Services Research and Health Care Technology, National Library of Medicine, Bethesda, MD, liz.amos@nih.gov; Research Data Informationist, NIH Library, National Institutes of Health, Bethesda, MD, lisa.federer@nih.gov; Research and Education Informationist, MUSC Libraries, Medical University of South Carolina, Charleston, SC, loganay@musc.edu; Retired, Formerly Director of Digital Data Curation Strategies, Lister Hill Library of the Health Sciences, University of Alabama, Birmingham, AL, tscott@uab.edu

## Abstract

As librarians are generally advocates of open access and data sharing, it is a bit surprising that peer-reviewed journals in the field of librarianship have been slow to adopt data sharing policies. Starting October 1, 2019, the *Journal of the Medical Library Association (JMLA)* is taking a step forward and implementing a firm data sharing policy to increase the rigor and reproducibility of published research, enable data reuse, and promote open science. This editorial explains the data sharing policy, describes how compliance with the policy will fit into the journal’s workflow, and provides further guidance for preparing for data sharing.

Many biomedical journals have instituted data sharing policies that require or encourage authors to make the data underlying the results described in their articles available to others to increase the rigor and reproducibility of the research [1]. Data sharing can also be beneficial to authors, who may obtain scholarly credit for citable datasets appearing on their curricula vitae, receive more citations of their journal articles, and encounter new opportunities for collaborating with other researchers [2, 3].

Because librarians are generally advocates of open access and data sharing, it is a bit surprising that peer-reviewed journals in the field have been slow to adopt data sharing policies. Some library journals, including the *Journal of Librarianship and Scholarly Communication* and *Journal of eScience Librarianship*, encourage authors to make their data available to others but do not make it a requirement. Because the *Journal of the Medical Library Association (JMLA)* editorial team thinks it is time to “practice what we preach” [4], the *JMLA* is taking a step forward and implementing a firm data sharing policy to increase the rigor and reproducibility of published research, enable data reuse, and promote open science.

## THE *JOURNAL OF THE MEDICAL LIBRARY ASSOCIATION (JMLA)* DATA SHARING POLICY

Starting October 1, 2019, authors of Original Investigation and Case Report manuscripts are required to deposit the de-identified data associated with their manuscripts in a repository and include a “Data Availability Statement” in their manuscripts describing where and how the data can be accessed. This data sharing policy was carefully developed by a *JMLA* working group [4] by soliciting feedback from authors of recently published *JMLA* articles, reviewing the data sharing policies of other journals, tracking the progress of the Research Data Alliance Data Policy Standardisation and Implementation Interest Group [5], and seeking feedback from the Medical Library Association (MLA) Board of Directors and other key constituents.

The *JMLA* defines “data” as the digital materials underlying the results described in the manuscript, including spreadsheets, text files, interview recordings or transcripts, images, videos, output from statistical software, or computer code or scripts. Digital materials supporting study methodology (e.g., survey instruments, interview questions, assessment tools) may be included as components of the dataset; however, survey instruments must also be provided as an appendix to be published alongside the article, as per existing *JMLA* guidelines.

Shared data should be appropriately de-identified to prevent revealing the identity of study participants. MLA, the *JMLA*, and individual members of the *JMLA* editorial team are not liable for any harm or damage resulting from the insufficient de-identification of data associated with *JMLA* articles.

Data can be placed in any repository that makes data publicly available and provides a unique persistent identifier. These can include institutional repositories, general repositories (e.g., Figshare, Open Science Framework, Zenodo, Dryad, Harvard Dataverse, OpenICPSR), or discipline-specific repositories that accept data of a particular format or in a particular domain. Repositories that allow restricted data access to other researchers who meet certain conditions are also acceptable. When possible, authors are encouraged to apply a license that is at least as permissive as a Creative Commons Attribution (CC BY) license to the data.

A brief “Data Availability Statement” should be placed at the end of the main text before the references. It should indicate the location of the data (e.g., name of repository); provide a hyperlinked unique persistent identifier, such as a digital object identifier (DOI), accession number, or persistent uniform resource locator (PURL); and describe any instructions for accessing the data, if applicable. If the manuscript does not describe the collection or analysis of data, which may be the case for some Case Reports, this should be stated in the “Data Availability Statement.” Statements akin to “data are available by request” or “data are reported in the manuscript” are not acceptable.

The *JMLA* editorial team expects authors to share at least the minimum amount of data needed to reproduce the results described in their manuscripts. Exactly what constitutes a “minimal dataset” will differ for each study and will largely be left to the authors’ discretion. For example, in cases in which it is not practical or appropriate to share full-length audio files or transcripts from interviews or focus groups, sharing the qualitative coding data may be adequate. Authors are encouraged to use open data formats [6] and to provide description or documentation (e.g., data dictionaries, codebooks, readme files) that allows others to understand the content and context of data files.

Exceptions to this policy will be made in rare cases in which de-identified data cannot be shared due to their proprietary nature or participant privacy concerns. Authors are expected to share only data that they are legally authorized to distribute. If the data are not owned by the authors, the data source and contact information should be noted in the “Data Availability Statement.”

## *JMLA* WORKFLOW

### Submission

The manuscript must include a “Data Availability Statement” at the time of submission. To protect the identity of authors during peer review and guard against the premature release of data, placeholder text can stand in for the repository name and persistent identifier. The *JMLA* editor will check that the submitted manuscript contains an appropriate “Data Availability Statement”; if not, the manuscript will be returned to the authors for correction before being sent out for review.

### Review

Due to several challenging aspects of the peer review of data [7], manuscript reviewers will not be explicitly asked to evaluate the data. If reviewers feel it is necessary to see the data to permit a complete evaluation of the study, they may request access to the data during the review period. In such cases, access to the data will be mediated by the *JMLA* editor.

### Acceptance

If the manuscript is accepted, the final version of the manuscript must contain a complete “Data Availability Statement” that includes a functional hyperlinked persistent identifier before it is sent for publication. Authors can opt to embargo their data in their chosen repositories until the anticipated date of article publication (which will be communicated by the *JMLA* editor upon or soon after acceptance). In that case, the hyperlinked persistent identifier should lead to a landing page containing basic dataset information (e.g., title, authors, description), although access to the data files themselves is not yet permitted. The *JMLA* editor will generally not scrutinize the contents of data files but will check that the nature of the data files in the repository appears to be congruent with the data collected for and analyzed in the study.

## FURTHER GUIDANCE

If you have never publicly shared your data before, you may feel the need for additional guidance on issues such as gaining institutional review board (IRB) approval for data sharing (including creating suitable informed consent forms for participants), de-identifying data, preparing documentation, selecting file types, or choosing an appropriate repository. Luckily, our profession is rife with experts in research data management (e.g., members of the MLA Data Special Interest Group) who have created or authored a wealth of LibGuides (e.g., University of Pennsylvania Libraries [8], Washington University Libraries [9], Johns Hopkins Libraries [10]); books and articles (e.g., Federer [11], Barbrow et al. [12]); and online training modules (e.g., Read and Surkis [13], Johns Hopkins Libraries [14], MIT Libraries [15], University of Massachusetts Medical School Lamar Soutter Library [16]) in this area.

The Association for Information Science and Technology (ASIS&T) webinar “Best Practices for Data Sharing and Deposit for Librarian Authors” [17] (with openly available slides and transcript [18]) is particularly helpful. You could also consult directly with a data librarian at your institution or refer to the many excellent guides to data sharing resources from other scholarly research organizations, including data repositories (e.g., Inter-university Consortium for Political and Social Research [19], Dryad [20], Databrary [21]). And of course, you may contact the *JMLA* editor (*JMLA*@journals.pitt.edu) with specific questions or concerns about your compliance with the data sharing policy.

## CONCLUSIONS

Recognizing that data sharing requires extra time and effort from authors [22] and that ensuring authors’ compliance with a data policy adds time to the journal’s editorial workflow [23], the *JMLA* working group sought to develop a data sharing policy that imposes a reasonably minimal burden on both authors and editors. We stress that we do not intend the new data sharing policy to be a barrier to publishing in the *JMLA*. Rather, we ask authors to share the data underlying their results to the extent that is practical, useful, and ethical, and if data cannot be shared, to be transparent about the reasons. We deeply appreciate the quality of our authors’ work and thank you in advance for joining us in this endeavor to further enhance the strength and transparency of research in health sciences librarianship and adjacent fields.

## 

**Katherine G. Akers**, *JMLA*@journals.pitt.edu, https://orcid.org/0000-0002-4578-6575, Editor-in-Chief, *Journal of the Medical Library Association*

**Kevin B. Read**, kevin.read@nyumc.org, https://orcid.org/0000-0002-7511-9036, Data Services Librarian and Data Discovery Lead, NYU Health Sciences Library, New York University School of Medicine, New York, NY

**Liz Amos**, liz.amos@nih.gov, Librarian, National Information Center on Health Services Research and Health Care Technology, National Library of Medicine, Bethesda, MD

**Lisa M. Federer, AHIP**, lisa.federer@nih.gov, https://orcid.org/0000-0001-5732-5285, Research Data Informationist, NIH Library, National Institutes of Health, Bethesda, MD

**Ayaba Logan**, loganay@musc.edu, https://orcid.org/0000-0002-7430-6358, Research and Education Informationist, MUSC Libraries, Medical University of South Carolina, Charleston, SC

**T. Scott Plutchak, AHIP, FMLA**, tscott@uab.edu, https://orcid.org/0000-0003-4712-5233, Retired, Formerly Director of Digital Data Curation Strategies, Lister Hill Library of the Health Sciences, University of Alabama, Birmingham, AL
