# Correction

**DOI:** 10.5195/jmla.2020.892

**Published:** 2020-01-01

**VOLUME 107**

107(4) October, page 470 and page 471

Akers KG, Read KB, Liz Amos, Federer LM, Logan A, Plutchak TS. Announcing the Journal of the Medical Library Association’s data sharing policy [editorial]. J Med Libr Assoc. 2019 Jul;107(3):468–71. DOI: http://dx.doi.org/10.5195/jmla.2019.801.

Page 470: An acknowledgment is missing. The “Acknowledgments” should be:

We thank Ariel Deardorff and Erin Diane Foster, former MLA Data Special Interest Group conveners, for their help in informing MLA members about the *JMLA* data sharing policy. The contributions to this work by Liz Amos and Lisa Federer were provided with the support of the National Library of Medicine (NLM), National Institutes of Health.

Page 471: Author Lisa Federer’s affiliation information is incorrect. The author affiliation should be:

**Lisa M. Federer, AHIP,** lisa.federer@nih.gov, https://orcid.org/0000-0001-5732-5285, Data Science and Open Science Librarian, National Library of Medicine, National Institutes of Health, Bethesda, MD

